# A Review of COVID-19 Modelling Strategies in Three Countries to Develop a Research Framework for Regional Areas

**DOI:** 10.3390/v13112185

**Published:** 2021-10-29

**Authors:** Azizur Rahman, Md Abdul Kuddus, Ryan H. L. Ip, Michael Bewong

**Affiliations:** 1 School of Computing, Mathematics and Engineering, Charles Sturt University, Wagga Wagga, NSW 2678, Australia; mdabdul.kuddus@my.jcu.edu.au (M.A.K.); hoip@csu.edu.au (R.H.L.I.); mbewong@csu.edu.au (M.B.); 2 Institute for Land, Water and Society (ILWS), Charles Sturt University, Albury, NSW 2640, Australia; 3 Australian Institute of Tropical Health and Medicine, James Cook University, Townsville, QLD 4814, Australia; 4 Department of Mathematics, University of Rajshahi, Rajshahi 6205, Bangladesh

**Keywords:** COVID-19, models, different settings, intervention strategies, NSW

## Abstract

At the end of December 2019, an outbreak of COVID-19 occurred in Wuhan city, China. Modelling plays a crucial role in developing a strategy to prevent a disease outbreak from spreading around the globe. Models have contributed to the perspicacity of epidemiological variations between and within nations and the planning of desired control strategies. In this paper, a literature review was conducted to summarise knowledge about COVID-19 disease modelling in three countries—China, the UK and Australia—to develop a robust research framework for the regional areas that are urban and rural health districts of New South Wales, Australia. In different aspects of modelling, summarising disease and intervention strategies can help policymakers control the outbreak of COVID-19 and may motivate modelling disease-related research at a finer level of regional geospatial scales in the future.

## 1. Introduction

Over the last few decades, the world faced a massive challenge in controlling infectious disease outbreaks in several areas [1]. Recently, a new infectious disease, SARS-CoV-2 named COVID-19, a virus of *coronaviridae* family and genus beta coronavirus, has emerged globally, and almost all countries and territories are now fighting against this newly appeared infectious disease [2]. The Municipal Commission in Wuhan, China, reported a cluster of pneumonia cases that had an unfamiliar etiology on 12th December 2019. COVID-19 was first identified in Wuhan city, Hubei Province of China, on 31st December 2019, and it spread so fast that within only five months, nearly two million people were infected in 185 countries around the world [3]. On 11th March 2020, the World Health Organization (WHO) announced the transmission of COVID-19 as a global pandemic because of the rapid increment of its infection rate [4]. Following SARS-CoV, which originated in China in 2003, and MERS-CoV, which originated in Saudi Arabia in 2013, SARS-CoV-2 seems to have become the third most significant public health concern of its type. The current fatality rate for COVID-19 cases is about 3.4%, significantly less than SARS and MERS, but potentially higher than those reported for endemic human non-SARS CoV infections [5].

The number of cases quickly rose to 44, with 11 of these patients in severe condition on 3rd January 2020. The COVID-19 virus spread across mainland China with over 30 thousand confirmed cases and over 600 deaths within only one month [6]. The World Health Organization (WHO) published an online resource that presented countries with guidance on detecting, testing and controlling possible cases on 10th January 2020 [7]. The first case outside of China was reported on 13 January 2020. Then, by 11th March 2020, the WHO declared COVID-19 to be a pandemic, based on its fast spread outside China. As of 11th November 2020, over 51.3 million people have been infected globally, with a 2.5% death rate [8]. Currently, almost 47.7% of the total global infections are in three countries—the United States (US), India, and Brazil. Together, deaths in these countries make up around 41.7% of global deaths [8]. According to the Worldometer estimation, up to the date 20th July 2021, nearly 191.7 million people have been identified as infected, with more than 4 million deaths, and about 174.5 million individuals have recovered in 213 countries and territories around the globe [9].

In the US, state and local governments, following the Center for Disease Control (CDC) guidance, started monitoring all individuals who had been in close proximity with confirmed COVID-19 cases. As a result, by 26th February 2020, 12 travel-related positive cases and three positive cases with no travel history were documented [10]. Specifically, the latter category of infections was a cause for concern since it indicated a significantly higher presence of the virus in the United States. In worldwide COVID-19 deaths, the US has been severely burdened by the disease and it alone accounts for about 18.9% of the global deaths, followed by Brazil and India with about 12.8% and 10.0% of global deaths, respectively [4].

The first cases of COVID-19 were linked to a live animal market in Wuhan, China [11]; however, the current rapid spread is via human-to-human transmission. Once infected, the individual will first undergo a period without visible clinical symptoms, called a latent SARS-CoV-2 infection. People with latent SARS-CoV-2 can become infectious one to two days before the onset of symptoms and continue to be infectious up to seven days after that [12]. Therefore, after a certain period, the latent SARS-CoV-2 infection progresses to an active COVID-19 infection. The disease spreads quickly from a person with active COVID-19 infection to another person when the infectious and susceptible persons are close [13]. The spread of COVID-19 depends on the length of exposure of susceptible people to the infected person [14]. It is, in turn, dependent on many factors, such as the crowdedness of the environment, any super-spreading events, the prevailing climatic conditions and the immune status of the exposed individual [15].

Despite extensive epidemiological research on various coronaviruses, there are still many unknowns about this new disease. It is thought that COVID-19 primarily spreads via respiratory droplets and aerosol and has an incubation period of up to 14 days, with symptom onset generally occurring at around days 5–6, similar to SARS-CoV, the cause of the severe acute respiratory syndrome (SARS) epidemic in 2002 [16,17,18]. However, unlike SARS-CoV, which resulted in high viral loads in the lower respiratory tract and led to viral shedding with symptom onset, SARS-CoV-2 has been shown to result in viral shedding due to asymptomatic infection from the upper respiratory tract and making it problematic to organisation preventative procedures that depend on symptomatology [6,19]. As a result, it led to an extreme contact rate from infectious persons to susceptible individuals, and while SARS was basically under control within eight months, the nature of COVID-19 is resembled differently due to the several variants [20]. COVID-19 has various signs and symptoms, varying from a mild cough and fever to a shortness of breath, pain, and even anosmia [21]. The disease is also severely prevalent, with most affected persons being asymptomatic or presenting only mild symptoms. However, the other critical forms of COVID-19 require hospitalisations and, in many cases, prolonged intubations. Treatment for the COVID-19 generally focused on supportive capacities, with only limited antiviral medicine (and announced vaccines in all nations that are open or ready for extensive use to remarkably reduce the number of people dying from COVID-19 through vaccination), presenting some promise at that moment [22,23,24,25,26].

A recent study on risk factors conducted by the Oxford Royal College of General Practitioners Research and the Surveillance Centre primary care network investigated severe disease combined infection rate and disease rate and showed a higher probability of infection for older people, men, people of ethnicity other than white, as well as people from areas with a higher socio-economically deprivation or population density [27]. In addition, initial studies showed COVID-19 to be associated with older age, ethnicity, high population density, and comorbidities such as respiratory infections, hypertension, diabetes, and cardiovascular diseases [19,21,28,29,30]. Notwithstanding significant improvements in science and technology, our perception of the pathogenesis of COVID-19 still seems to be rudimentary, with new (and sometimes conflicting) data emerging almost daily to address the pandemic more efficiently and a race to possible intervention strategy selections.

Modelling has been used as a tool to address gaps in knowledge and to inform health policies for the prevention and control of COVID-19 [31,32,33,34]. Currently, researchers have developed different types of modelling approaches to estimate the relationship between COVID-19 and various risk factors in different sociodemographic and geospatial settings [21,33,35,36,37]. In addition, modelling studies also explore the impact of different intervention strategies to identify the most effective ones. In this study, we carry out a literature review on COVID-19 and infectious disease modelling strategies to develop a robust research framework for the regional areas of New South Wales (NSW), Australia. We believe this may help improve the control strategy for COVID-19 epidemics at the regional level in NSW, and the prospective modelling outcomes will be helpful to decision-makers.

## 2. Modelling Experience from three Countries for COVID-19

In this section, we appraise different modelling strategies used for the COVID-19 outbreak in three countries—China, the UK, and Australia. Within Australia, we will focus on the transmission dynamics modelling approach considered in NSW.

### 2.1. Models with Single and Multiple Interventions

A mathematical model is an essential tool to determine which combination interventions would be most effective for reducing the outbreak of COVID-19. Prem et al. [34] developed a modified SEIR model to investigate the impact of physical distancing and population mixing on the progression of COVID-19 in Wuhan, China. In this study, the authors applied synthetic location-specific contact patterns in Wuhan and adjusted these for school closures, extended workplace closures, and decreasing mixing in the general population. They also considered predicting the impact of lifting control measures by permitting people to return to work in their offices. This study found that physical distancing measures were the most useful for controlling COVID-19 in Wuhan. However, implementing physical distancing measures produced varying results, with the duration of infectiousness and the adaptation of school and workplace closures during COVID-19 outbreaks. This study suggests that the premature and sudden lifting of restrictions could lead to a secondary outbreak. Nevertheless, the risk of a secondary outbreak could be minimised or controlled by relaxing restrictions systematically. The limitations of this study are statistical uncertainties about measures of the basic reproduction number and the continuation of infectiousness.

Most of the mathematical modelling studies focus on the transmission dynamics of COVID-19 and do not consider the changing epidemiology and temporal and spatial transmission heterogeneity. Hou et al. [38] developed a modified multi-stage SEIR model to describe the transmission dynamics of COVID-19 in Wuhan at different spatio-temporal scales. In this study, the authors consider the variation in infectivity and introduce the control, the basic reproduction number, by assuming the exposed population to be infectious and simulate the future spread of COVID-19 across Wuhan. The authors also built a novel source-tracing algorithm to infer the initial exposed number of individuals and to estimate the number of infections during the epidemic. The significant findings of this study are that the spatial patterns of COVID-19 spread are heterogeneous, and the infectivity is significantly more remarkable for the exposed population than the infectious population. However, in this study, the predicted exposed population is much greater than the officially reported size of the infectious population in Wuhan.

Due to the insufficient number of COVID-19 vaccines in the early stage, in many countries, lockdown is one of the most effective measures to control the spread of infection and to evaluate the influence of non-pharmaceutical interventions, including the reopening of schools and workplaces, as well as household contacts, and the broader relaxation of physical distancing. Panovaka-Griffiths et al. [39] develop a stochastic individual-based model for the transmission dynamics of COVID-19 in the UK to estimate the impact of school reopening strategies and contact tracing–testing scenarios. The results showed that increasing testing levels and effective contact tracing coupled with isolation might control COVID-19 in the UK. However, without raising testing levels and widespread contact tracing, the reopening of schools together with the gradual relaxing of lockdown measures are likely to cause secondary outbreaks of COVID-19. This study suggests that for preventing secondary spikes in COVID-19 in the UK, the relaxation of physical distancing such as the reopening of schools must be followed by large-scale, effective contact tracing, supported by isolation and the testing of symptomatic individuals [39].

Despite the first confirmed case of COVID-19 in the UK occurring on 30th January 2020, the UK government waited until lab-confirmed cases reached 11,080 before initiating a lockdown on 24th March [40]. How and when to make public health decisions during epidemics are challenging questions to answer. The appropriate policy response should be based on scientific evidence, which depends on good data and modelling. Modelling is the most effective way of measuring and controlling the current outbreak of COVID-19. The critical parameter for explaining the spread of COVID-19 is the basic reproduction number, which is the expected number of secondary cases caused by a single infectious individual introduced into a susceptible population. If the basic reproduction number is less than one, the disease is endemic; otherwise, it is an epidemic. In looking at the effect of the basic reproduction number on the dynamics of the outbreak of COVID-19 in the UK, Wang et al. [41] considered the SIR and SEIR model. Here, the authors defined four types of populations; susceptible (S)—those who are not in contact with the virus but might be infected as a result of transmission from an infected individual; Exposed (E)—those who are infected but not infectious; Infected (I)—those who are infected and infectious; Removed (R)—those who were previously infected but are now free of the disease. The results showed that the basic reproduction number plays a crucial role in explaining the dynamics of the outbreak of COVID-19 in the UK, but due to the novel nature of COVID-19, there is still a challenge to evaluate the epidemiological implications. Therefore, further research is urgently required to fill the gaps.

COVID-19 spreads quickly from one person with the virus to another person when the infectious person coughs and the susceptible person comes into physical contact [13]. Stutt et al. [42] developed a mathematical model to show the effect of wearing facemasks with or without lockdown times on the transmission dynamics of COVID-19 in the UK. The results showed that when the public adopts wearing facemasks most of the time, the effective reproduction number can be reduced to below one, leading towards the elimination of epidemic spread. Furthermore, when lockdown times are implemented in combination with facemask use, there is a lesser spread of the disease, and the secondary peak is not as high. This study suggested that a combination of strategies, including wearing facemasks and social distancing or lockdowns, may constitute a satisfactory policy for controlling COVID-19.

COVID-19 has placed significant extra pressure on hospital intensive care services in many countries, including Australia [43]. Mathematical modelling can provide important insights into the likely cause of the epidemic—these insights are valuable for the intensive care services during the epidemic. Adekunle et al. [36] developed a stochastic metapopulation model to describe the effect of travel bans imposed globally and within Australia on international flight travel volumes. The results showed that travel bans on international passengers arriving from different countries, including Iran, Italy and South Korea, had no significant impact on decreasing the outbreak of COVID-19 cases. However, in the case of a ban on travellers from China, it did have a significant impact. The authors mentioned that one reason for this was that the prevalence of the disease in countries like Iran, Italy and South Korea was lower than in China, and Italy had previously implemented a lockdown by the time Australia implemented restrictions on travellers coming from Italy. Thus, they suggested that the travel ban is very efficient in delaying the extensive transmission of COVID-19. A similar conclusion was drawn by Ip et al. [44] who evaluated various mitigation policies implemented by the state and federal governments of Australia using a generalised space–time autoregressive model. They found that both international and interstate border controls helped to reduce the number of new COVID-19 cases in Australia.

Kang et al. [6] explained the spatio-temporal pattern and explored the spatial relationship of the COVID-19 epidemic in mainland China. This study found that most of the models, except medical-care-based connection models, showed a significant spatial relationship of COVID-19 infections, which means that the management of the spatial spread in the early stage of COVID-19 is very significant for the control of the further transmission. However, although this study has incorporated the spatial aspect of COVID-19, it has some limitations. Firstly, this study did not take into account the number of suspected cases. Therefore, it is a challenge to understand the spatio-temporal transmission of COVID-19. Secondly, this study did not incorporate the urban–rural connection, which might have an important impact on transmission. Therefore, further research is needed to include the most critical factors and to explore the spatial spread of COVID-19.

Costantino et al. [45] developed a deterministic model to further explore the effectiveness of full and partial travel bans in Australia for travellers from China against the spread of COVID-19. They modelled three basic scenarios—no ban, the current ban, followed by a full or partial lifting to examine the influence of travel bans on the dynamics of COVID-19 outbreak control. Moreover, they used COVID-19 incidence data from China and details of passenger flights between China and Australia during and after the outbreak in China, obtained from incoming passenger arrival record cards. The results show that without a travel ban, an increase of more than 2000 cases and around 400 deaths would have occurred. The complete travel ban decreased the number of cases by more than 86%, while the partial travel ban reduced the number of cases by 50%. These figures indicate the efficacy of policy decisions. This study suggests that imposing travel restrictions with a country (China) experiencing an epidemic peak is highly effective. Further tabulated information of the key literature review on COVID-19 modelling in China, the UK, and Australia is summarised in Table 1, which follows.

### 2.2. Models with Age Structure and Vaccination

Age is one of the significant factors which can influence the occurrence and severity of the COVID-19 disease. Chang et al. [59] developed an agent-based model for transmission dynamics of the ongoing COVID-19 outbreak in Australia. The authors applied the model to compare several intervention strategies, including travel restrictions, case isolation, school closures, social distancing, and home quarantine. The results showed that the rate of symptomatic cases in children is one-fifth of the rate for adults. This study also shows that the intervention of school closures alone was not effective unless coupled with a high level of social distancing. Therefore, the authors asserted that the combination of social distancing with effective isolation and international travel restrictions was the most effective way to control the outbreak of COVID-19.

Vaccination is often considered the best way to prevent or control outbreaks of infectious diseases including COVID-19 [64]. In addition, in the cases of some infectious diseases, there is no specific treatment except vaccination. Although the exploration of vaccines for COVID-19 was a great challenge, different types of vaccines are now available to combat the spread of COVID-19. The European Medicines Agency and the Italian Medicines Agency have approved Pfizer, Moderna, AstraZeneca AZD1222 and J&J Ad26.COV2.S on 13th March 2021 [65]. 

Table 2 presents a tabulated summary of the current models that include the vaccination strategies specifically focused on China, the UK, and Australia. For instance, McBryde et al. (2021) developed a COVID-19 model with a vaccination to explore the direct and indirect effects of vaccination by vaccine type, age strategy, and coverage in Australia [66]. The model incorporated some crucial factors, including age-specific mixing, infectiousness, susceptibility and severity, to examine the epidemic under different intervention scenarios. The authors found that the current mixed program, including vaccination with AstraZeneca and Pfizer, would not achieve herd immunity unless 85% of Australia is covered, including 5–16 years of age and considering the effective reproduction number for Delta variant is 5. However, when the value of the effective reproduction number is 3, the mixed program can achieve herd immunity at 60–70% coverage without vaccinating 5–15 years of age. The general finding of this study was that vaccination can prevent 85% of death compared to without vaccination [66]. 

In 2021, with numerous vaccines becoming available in Australia, Maclntyre et al. (2021) developed a compartmental COVID-19 model to explore the vaccine’s effectiveness for target groups, including health workers, young people and older adults, and mass vaccination in NSW Australia [67]. For the target group, results showed that health worker vaccination is necessary for health system resilience. Furthermore, age-based policies with restricted doses of the vaccine can reduce a small amount of infections, but vaccinating older people reduces the prevalence of death. On the other hand, mass vaccination, including 66% of the NSW population, can achieve herd immunity. However, this study also found that slower vaccination rates can lead to a prolonging of the COVID-19 pandemic, and a higher number of cases and deaths in the population [67].

Besides, to measure the optimal vaccine prioritisation of COVID-19 transmission, Han et al. (2011) developed a data-driven mechanistic model in China [68]. In this model, they considered 17 age groups and divided the population into five compartments: the unvaccinated susceptible population (S); persons who received at least the first dose of vaccine but have yet to develop protection (V); persons who received the second dose of the vaccine but failed in protection (U); infectious individuals including asymptomatic and symptomatic infections (I); and recovered or immune individuals (R). The result showed that a time-varying vaccination program for the different age groups is the most effective means of reducing deaths and infections. Furthermore, this study recommended that, to minimise the number of deaths and ICU admissions, people over 65 years of age should be vaccinated before moving to other groups such as younger and middle-aged people. Finally, the early phase of high vaccination capacity is the key to achieving significant success of policy measures and implementations [68]. 

Moreover, a mathematical model with different age groups in the UK was proposed by Moore et al. (2021) to investigate different COVID-19 vaccination scenarios and the age-specific vaccine efficacy [69]. A modified SEIR-type model was considered with a force of infection determined by age-dependent social contact matrices. The authors assumed that the new secondary infections increase due to the first infections within a household. However, the secondary household contacts were to be quarantined and subsequently performed no additional role for the outbreak of COVID-19. The result showed that vaccination is the most effective for the elderly and vulnerable population, which helped reduce the number of deaths and healthcare demands [69]. Modelling vaccination with non-pharmaceutical interventions is necessary to investigate significant variations in behaviours associated with COVID-19 prevention, detection and treatment than a single intervention. Furthermore, Moore et al. [70] proposed another age-structured model-integrated two-dose vaccination and non-pharmaceutical interventions in the UK. The finding showed that vaccination alone is not sufficient to contain the outbreak of COVID-19. In the absence of non-pharmaceutical interventions, the vaccine will prevent 85% of infections in the population. Combining vaccination and non-pharmaceutical interventions can eliminate the COVID-19 outbreak in the UK [70].

Statistically, modelling plays a vital role in efforts that focus on predicting, assessing, and controlling potential outbreaks of different kinds of infectious diseases. Modelling can also be used to explore the contagious disease dynamics that impact numerous variables ranging from the micro host–pathogen level to host-to-host interactions and dominant ecological, social, economic, and geographical factors across the globe. Additionally, Table 3 discusses some key literature for different infectious disease modelling approaches and their control strategies. For instance, Kanyiri et al. (2018) provide modelling results of the transmission dynamics of influenza by incorporating the aspect of drug resistance and using dynamical systems and sensitivity analysis [71]. Overall, the findings of Table 3 studies reveal some consistencies and disparities between the modelling tools and techniques, as well as the diseases and the nature of infections. Indeed, the knowledge of these modelling approaches would help develop a contemporary and robust research framework, which may specifically focus on different spatial levels within a region. Location-specific knowledge is required to develop an appropriate model for a particular area such as regional areas in NSW, Australia.

## 3. Developing Models with a Regional Focus

COVID-19 poses a significant challenge for the government healthcare system in regional areas of NSW. One of the most significant challenges is the demand for hospitals to treat critically ill COVID-19 patients [60]. Current knowledge from the outbreak in Italy suggests that a severe demand for intensive care support can occur at the peak of an epidemic. The shortage of intensive care support often leads to preventable deaths due to the lack of accessible intensive care units (ICU) and healthcare workers [81]. The epidemic trajectory of COVID-19 in NSW seems delayed by many weeks compared to several states, including Victoria, due to the travel bans implemented at the beginning of the epidemic. The situation is changing very quickly, and NSW government policy has recently focussed on prevention rather than lockdowns or eliminating COVID-19 infection from the community [60]. Nonetheless, unless an effective vaccine is produced, it seems possible that the outbreak of this disease will transmit quickly within the general population [82]. The effectiveness of current and prospective non-pharmaceutical intervention strategies, including social distancing, is unpredictable or highly reliant on the extent to which they are implemented.

Mathematical modelling is one of the most effective ways to gain insights into the dynamics of an epidemic and to assist in the allocation of resources, including intensive care resources, during different stages of the pandemic. Fox et al. [60] developed a modified SEIR model to estimate hospitalised cases and ICU cases per 100,000 population in NSW. This study considers two scenarios; one is no intervention within a basic reproduction number of 2.4, and the other is social distancing strategies leading to a basic reproduction number of 1.6. The results showed that without social distancing measures, the peak of the COVID-19 cases for hospitalisation would be 450 per 100,000, with about 150 people needing intensive care. According to the scenario without intervention, the outbreak infection peak would be late June and hospital usage in early July. Under the second scenario with social distancing, around 180 people would be hospitalised per 100,000, with 65 people needing intensive care. The outbreak will move to early October, and peak ICU usage will move to mid-November. The authors suggested that the social distancing intervention strategy would be partially effective for the delay of the epidemic peak by around 12 weeks. However, this study did not estimate the effect of suppression strategies, which would reduce the peak of ICU demand. Therefore, further modelling is required to explore the impact of suppression strategies at the time of the epidemic in NSW, including on ICU demand. Such modelling strategies will help to notify the public concerning the timing, severity, and continuation of mitigation policies.

Weather variables including temperature, humidity and rainfall are critical determinants for the outbreak of COVID-19 in NSW [83] and other states and countries [84]. To explore the association between meteorological variables and the number of COVID-19 cases, Ward et al. [83] used a time series analysis in NSW. They used a multivariate generalised additive model (GAM) where a correlation matrix was used to select a weather variable to avoid multicollinearity in the analysis. The best model was selected based on the backward algorithm and the Akaike information criteria (AIC) value. Weather variables were analysed through a 14-day interval based on the incubation time, and the natural splines function with two degrees of freedom is used for the model trend and seasonality. The results showed that temperature and rainfall have no relationship with COVID-19 in NSW, while low temperature and low humidity are suitable for the survival and spread of the virus, because they dry out the mucous membrane, reduce the function of cilia and facilitate the spread of suspended matter in the atmosphere [84,85]. Some modelling studies suggested that lower temperatures may increase the number of COVID-19 cases [84,86]. Therefore, more research is needed to explore the association between temperature and the number of COVID-19 cases.

**Figure 1:**
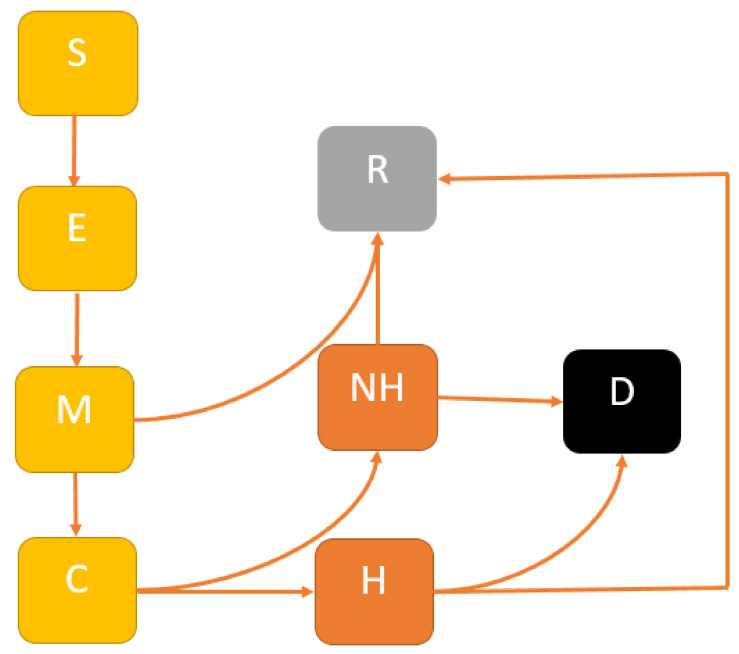
Extended SEIR model structure: The population is divided into the following six classes: susceptible, exposed (and not yet symptomatic), infectious (symptomatic), i.e., mild (mild or moderate symptom) and critical (severe symptoms), death and recovered (i.e., isolation, recovered, or otherwise non-infectious).

In the future, we propose to develop a comprehensive model of COVID-19 transmission dynamics over time to infer the impact of mitigation, suppression and multiple interventions and their cost-effective analysis for controlling COVID-19 outbreaks in NSW. We will develop a modified SEIR model to account for the following mutually exclusive compartments: Susceptible $S\left(t\right)$, uninfected individuals who are susceptible to the COVID-19 infection; Exposed $E\left(t\right)$, representing those who are infected and have not yet developed active COVID-19; Infectious $I\left(t\right)$, comprising individuals with active COVID-19; the Recovered $R\left(t\right)$, who were previously infected and successfully treated, or death $D\left(t\right)$. For estimating healthcare needs, we will categorise the infectious group into two sub-cases: Mild $M\left(t\right)$ and Critical $C\left(t\right)$; where Mild cases do not require hospital beds; and Critical cases need hospital beds. A flow diagram of our proposed model is presented in Figure 1.

To the best of our knowledge, in previous modelling studies, many mathematical models have been investigated, focusing on mysterious transmission dynamics of COVID-19 using different types of intervention strategies. However, none of them have used a cost-effective analysis for the economy in NSW, Australia. This model will consider a set of non-linear differential equations and will distinguish two essential features—the direct link between the Exposed and Recovered population and the practical healthcare demand resulting from the separation of infections into mild and critical cases. First, we will use a next-generation matrix to determine the basic reproduction number $R_{0}$ of COVID-19, where $R_{0}$ is the estimated number of secondary cases produced by single infectious cases and exclusively the susceptible population. Then, to supplement and validate the model structure, we will calibrate the number of cases from the COVID-19 data in NSW. Following this, we will perform a sensitivity analysis to explore the impact of parameters on the model outcomes. Finally, we will incorporate the economic compartment into our proposed model to explore the financial consequences of different interventions and their impact on the dynamics of COVID-19 in NSW, Australia.

## 4. Conclusions

COVID-19 has had more attention from the government and media than any previous infectious disease, including influenza. Modelling studies can contribute to developing novel control methods, improving computational tools, and public data sharing. For example, modelling studies strongly advised border closures, and China first imposed an internal travel lockdown on Wuhan, which delayed the epidemic peak of COVID-19 within China but had a more significant impact on other countries [35,87,88]. Statistical modelling has also projected the shifting of outbreaks from one country to another, based on these locations’ connectedness [89].

Age is a significant risk factor that can increase the severity of the outbreak of COVID-19. Mixing models can examine age-specific contact patterns and infection risk and use relative infectiousness [90]. Modelling studies have found that children are less likely to acquire an infection, and when infected, they are much less likely to show symptoms. This information will assist policymakers in strategy development. In addition, mixing models have showed that school lockdowns have a modest impact on COVID-19 transmission, encouraging authorities to re-open schools or to avoid school lockdowns completely [34,91].

Mathematical models can estimate the potential epidemic outbreak of COVID-19. One of the essential components for the modelling studies is the basic reproduction number, which is the expected secondary cases caused by a single infectious case in a susceptible population. Modelling studies have shown that implementing suppression (i.e., immediate lockdown) strategies will decrease the reproduction number to less than one, which means that the disease dies out gradually without the need to take any further action. Furthermore, any deficiencies in performing mitigation strategies will increase the risk of having a reproduction number greater than one, which indicates that the disease persists in the population, and governments need to take more actions to control the disease [92]. Intervention strategies and government-imposed constraints on human migration have started to decrease the spread. Models presenting variations in transmission rates over time have been influential tools, helping decision-makers to implement improvements in outbreak control within public health strategies [93].

It is well known that vaccines are very effective for infectious disease control [94,95]. Therefore, for the elimination of COVID-19, a vaccine is urgently needed for global-scale use. There are many clinical trials of COVID-19 vaccines underway, though a few countries claimed success in efficacy trials at their local or national scale. Modelling studies are beneficial in evaluating the effectiveness of vaccines within clinical trials and for reducing biases [96,97]. Modelling can also assist in evaluating the possible effectiveness of vaccination policies, including location-specific ring-vaccination, age-specific vaccination, and the socioeconomic and geopolitical advantages of vaccination. However, for COVID-19, the situation is even more challenging as the disease affects different age groups differently. There is also a greater risk of co-infection and mortality with other diseases, especially in the older age group. 

The information generated from the models of the COVID-19 pandemic allows collaborative involvement between decision-makers and researchers. Policymakers can provide researchers with a clear outlook of the policy settings, while researchers can construct models that assist in decision-making. Decision-makers can then plan the policy aims and the intervention strategies and should ideally build a setting where decision-makers and modellers work in combination on an ongoing basis.

Modelling studies may also perform a crucial role in expanding the scope of limited resources under discussion. For instance, a modelling study infers that UK health officials did not examine a policy that included testing due to a limited testing capacity [98]. Modellers also advise using suppression strategies in China rather than mitigation, as the results reduce exposure in China and reduce the number of global cases [99]. Modelling may also provide more optimal scenarios for different intervention strategies with significant benefits at a low cost. For example, in Australia, mitigation strategies are commonly considered rather than suppression strategies (except in Melbourne recently during the second wave of COVID-19 outbreaks) [61]. If modelling studies show that suppression strategies would provide better results, these actions can be implemented early in Australia, including in NSW. Our future application paper will consider this in the context of analysing epidemiological surveillance data to develop an optimal strategy to control COVID-19-type outbreaks in urban and rural health districts of NSW efficiently.

Non-pharmaceutical interventions and vaccination strategies are implemented to prevent and control COVID-19 in most countries in the world. Modelling can assess the potential impact of different interventions measures for mitigating the burden of COVID-19 across the globe [100]. Vaccination is the best way to prevent or control outbreaks of COVID-19. Mathematical models can examine the impact of vaccination on death if herd immunity is not achieved, and it also explores the direct effects of vaccination on reducing death are very good for which vaccines. Therefore, the steps for future research in modelling will be models with a combination of control strategies.

In this review, we have discussed some important COVID-19 models and have attempted to classify them by their structures (including some core assumptions). In addition, we summarise the model outcomes and distinctive features, including the impact of different intervention strategies and their cost, stability, and sensitivity analysis to identify the most impelling risk factors addressing model biases. In doing so, we have identified some open challenges and encouraging prospects for upcoming COVID-19 modelling-related research.

Finally, every study has its limitations. For future research, it is prudent to note those limitations that have posed a challenge to the findings of this study. This study’s specific limitation is the reliance on previously published research regarding mathematical modelling of COVID-19 in three countries, including Australia, China, and the UK, from 2019 to 2021. In addition, the quality of information obtained might not always be reliable, e.g., incidence, prevalence, health demand, etc., which may contaminate findings.

## Figures and Tables

**Table 1:** Summary of the key findings of some important literature about COVID-19 modelling in China, the UK, and Australia.

Countries	Author(s)	Research Aims	Methodology	Significant Findings	Strategies
China	Zhao and Chen [46]	To characterise the dynamics of COVID-19 and explicitly parameterise the intervention effects of control measures in China.	A Susceptible Un-quarantined, Quarantined infected, Confirmed infected (SUQC) model is applied to analyse the daily cases of COVID-19 outbreak in China.	The quarantine and control measures are effective in preventing the spread of COVID-19.	Quarantine and control measures.
China	Liu et al. [47]	To summarise and share the experience of controlling the spread of COVID-19 and provide effective recommendations to enable other countries to save lives.	A modified SEIR model is applied. It considered many influencing factors including spring festival, sealing off the city and construction of the fangcang shelter hospital.	Four different scenarios were investigated to capture different intervention practices. The combination of intervention measures is the only effective way to control the spread and not a single one of them can be omitted.	Seal off the city, enough medical resources, a combination of several interventions, authorities did nothing to control the epidemic.
China	Hao et al. [48]	To reconstruct the full-spectrum dynamics of COVID-19 between January 1, 2020 and March 8, 2020 across five periods marked by events and interventions based on 32,583 laboratory confirmed cases.	A modified susceptible-exposed-presymtomatic infectious-ascertained infectious-unascertained infectious-isolated-removed (SAPHIRE) SEIR model is applied and considered presymtomatic infectiousness, time-varying ascertainment rate, transmission rates and population movements.	Identified two key features of the outbreak: high covertness and high transmissibility. Found multi-pronged interventions had considerable positive effects on controlling the outbreak of COVID-19 and decreasing the reproduction number.	Presymtomatic infectiousness, time-varying ascertainment rate, transmission rates and population movements.
China	Wu et al. [49]	To estimate the clinical age specific severity, which requires properly adjusting for the case ascertainment rate and the delay between the onset of symptoms and death.	A SIR model is applied, which included the number of passengers and confirmed cases who returned to their countries from Wuhan on chartered flights.	Estimated the overall case, symptomatic case, fatality risk, and found that the risk of symptomatic infection increased with age.	Case ascertainment rate, symptoms onset and deaths.
China	Mizumoto et al. [50]	To investigate a link between the wet market and the early spread of COVID-19 in Wuhan, China.	A quantitative modelling framework was applied, which includes daily series of COVID-19 incidence to estimate the reproduction number for market to human and human to human transmission, the probability of reporting and the early effects on public health.	Found that the basic reproduction number of market to human transmission was lower than for human to human transmission. In contrast, the reporting rate for cases stemming from market to human transmission is 2–34 fold higher than that for cases stemming from human to human transmission, suggesting that contact history with the wet market plays an important role in identifying COVID-19 cases.	Wet market to human and human to human transmission.
China	Zhang et al. [51]	To analyse contact survey data for Wuhan and Shanghai before and during the outbreak and contact-tracing information from Hunan province.	A simple SIR model applied to show the impact of age, contact patterns, social distancing, susceptibility to infection for the dynamics of COVID-19 in Hunan province, China.	The results showed that children 0 to 14 years of age are less susceptible to COVID-19 infection than adults 15 to 64 years of age. However, individuals 65+ years of age are more susceptible to infection. Further, this study found that social distancing alone is sufficient to control COVID-19 in China.	Age, contact patterns, social distancing and susceptibility to infection.
China	Pang et al. [52]	To compute the basic reproduction number and analyse the disease free equilibrium as well as sensitivity analysis.	A modified SEIR model was used to explore the dynamics of COVID-19 in Wuhan, China and calculate the most important parameters.	The transmission rate is the most important parameter that can increase the severity of COVID-19 outbreak.	Transmission rate.
UK	Yang et al. [53]	To conduct a feasibility study for robustly estimating the number and distribution of infection, growth of death, peaks and lengths of COVID-19 breakouts by taking multiple interventions in the UK.	A modified SEIR model is used to infer the impact of mitigation, suppression and multiple rolling interventions for controlling the COVID-19 outbreak in the UK.	Rolling intervention is probably an optimal strategy to effectively and efficiently control COVID-19 outbreaks in the UK.	Mitigation, suppression.
UK	Davies et al. [54]	To assess the potential impact of different control measures for mitigating the burden of COVID-19 cases in the UK.	A stochastic age-structured transmission dynamic model is applied to explore the range of intervention scenarios and estimate the impact of varying adherence to interventions across countries.	Four base interventions including school closures, physical distancing, shielding of people aged 70 years or older and self-isolation were each likely to decrease the basic reproduction number but not sufficiently to prevent ICU demand from exceeding health service capacity. Intensive interventions with lockdown periods will need to be considered to prevent excessive health-care demand.	School closures, physical distancing, shielding of people aged 70 years or older and self-isolation.
UK	Booton et al. [55]	To develop a regional transmission dynamics model of COVID-19, for use in estimating the number of infections, deaths and required acute and intensive care (IC) beds in the south west of the UK.	A modified age-structured SEIR model to estimate cumulative cases and deaths and the impact of interventions.	Before any interventions, the basic reproduction number value is 2.6, with social distancing reducing this value to 2.3 and lockdowns/school closures further reducing the basic reproduction number to 0.6, which indicates that lockdowns/school closures are very effective interventions for controlling COVID-19.	Social distancing, lockdowns/school closures.
UK	Stutt et al. [43]	To estimate the impact of facemasks as a non-pharmaceutical intervention, especially in the setting where high-technology interventions including contact tracing or rapid case detection are not feasible.	A modified SEIR model is used to examine the dynamics of COVID-19 epidemics when facemasks are worn by the public, with or without imposed lockdowns.	The results revealed that when facemasks are used by the public all the time, the effective reproduction number can be decreased below 1, leading to the mitigation of epidemic spread. Further, with the combination of lockdowns and 100% facemask use, there is vastly less disease spread.	Lockdowns and facemasks.
UK	Rawson et al. [56]	To investigate the efficacy of two potential lockdown release strategies including ending quarantine and a re-integration approach.	A SEIR model is used to explore the gradual release strategy by allowing different fractions of lockdown.	Ending quarantine for the entire population simultaneously is a high-risk strategy; a gradual re-integration approach would be more reliable.	Lockdowns.
UK	Thompson [57]	To predict the effects of different non-pharmaceutical interventions.	A simple SIR model is used to demonstrate the principle that a reduction in transmission can delay and reduce the height of the epidemic peak under different non-pharmaceutical interventions.	The results revealed that lockdowns are more effective than other non-pharmaceutical interventions and need to be implemented immediately for controlling COVID-19 in the UK.	Lockdowns, school closures, social distancing, shielding of high-risk individuals and self-isolation.
UK	Peiliang and Li [58]	To predict the number of cases and estimate the basic reproduction number under different scenarios.	A modified SEIR model structure is used to explore the effect of time lag and the probability distribution of model states under different interventions.	Self-isolation can reduce the basic reproduction from 7 to 2 in the UK. Strict lockdowns and social distancing are effective interventions for reducing the basic reproduction number below 2.	Self-isolation, lockdowns and social distancing.
Australia	Chang et al. [59]	To compare several intervention strategies including restrictions on international travel, case isolation, home quarantine, social distancing and school closures.	An agent-based model is developed for a fine-grained computational simulation of the ongoing COVID-19 pandemic in Australia.	The results showed that school closures do not bring decisive benefits unless coupled with a high level of social distancing. Furthermore, a 90% level of social distancing is effective to control the COVID-19 within 13–14 weeks when coupled with effective case isolation and international travel restrictions.	International travel, case isolation, home quarantine, social distancing and school closures.
Australia	Fox et al. [60]	To explore the effect of varying the infection reproduction number, which can be reduced by effective social distancing measures at the peak of the epidemic.	A simple SEIR model is used, which includes household quarantine and social distancing.	The results showed that without social distancing, the number of people requiring hospitalisation in NSW will peak at 450 per 100,000 population and the number of individuals requiring critical care are at 150 per 100,000 population.	Household quarantine and social distancing.
Australia	Moss et al. [61]	To estimate the healthcare requirements for COVID-19 patients in the context of broader public health measures.	An age- and risk-stratified transmission model of COVID-19 infection is used to simulate an unmitigated epidemic in current estimates of transmissibility and severity.	The results showed that case isolation and contact quarantine alone will not be sufficient to constrain case presentations within a feasible level of expansion of health sector capacity. Social restrictions will need to be applied at some level during the epidemic.	Case isolation and contact quarantine.
Australia	Milne and Xie [62]	To evaluate a range of social distancing measures and to determine the most effective strategies to reduce the peak daily infection rate and consequential pressure on the healthcare system.	A transmission dynamics individual-based model is used to generate the rate of growth in cases, the magnitude of the epidemic peak and the outbreak duration.	The application of all four social distancing interventions including school closures, workplace non-attendance, increased case isolation and community contact reduction is highly effective for controlling COVID-19 in Australia.	School closures, workplace non-attendance, increased case isolation and community contact.
Australia	Costantino et al. [45]	To test the impact of travel bans on epidemic control in Australia.	An age-specific deterministic model is used to explore the impact of three travel ban scenarios.	The results showed that without travel bans the epidemic in Australia will continue for more than a year, partial travel is minimal and may be a policy option. Finally, travel restrictions are highly effective for controlling the outbreak of COVID-19 in Australia.	Travel restrictions.
Australia	Adekunle et al. [36]	To evaluate the effect of travel bans in the Australian context and predict the epidemic until May 2020.	A stochastic meta-population model was used. It categorises the global population into susceptible, exposed, infectious or recovered (SEIR) individuals.	The results showed that without travel bans Australia would have experienced local transmission as early as January 15 and possibly would have become the Pacific epicentre. Furthermore, having interventions in place can reduce the outbreak of local transmissions of COVID-19 in Australia.	Travel bans.
Australia	Price et al. [63]	To describe how the epidemic and public health response unfolded in Australia up to 13 April 2020.	A SEEIIR model is applied to estimate the time-varying effective reproduction number, which can be used for controlling COVID-19 in Australia.	The results showed that the effective reproduction number is likely below 1 in each Australian state since mid-March and forecast that hospital ward and intensive care unit occupancy would remain below capacity thresholds during the last two weeks of March.	Intensity and timing public health intervention.

**Table 2:** Some current models that include vaccination strategies in China, the UK, and Australia.

Countries	Author(s)	Model	Assumptions Implicit (and Explicit)	Applications in Predicting COVID-19	Policy Implications
Australia	McBryde et al. [66]	An individual based model with vaccination.	The model incorporates some important factors including age-specific mixing, infectiousness, susceptibility, and severity to examine the epidemic size under different intervention scenarios.	Predicting the impact of combination second doses vaccination strategies including AstraZeneca and Pfizer. Vaccination can prevent 85% of death compared with no vaccination.	Australia government can take immediate action to vaccinate all population.
Australia	Maclntyre et al. [67]	An age-structured deterministic compartmental model.	Includes target groups including health workers, young people and older adults as well as mass vaccination to explore the effectiveness of vaccine.	Results show that health worker vaccination is necessary for health system resilience. Mass vaccination which includes 66% of the NSW population can achieve the herd immunity. Slower rates of vaccination can lead to COVID-19 longer, higher cases and deaths in the population.	Must be vaccinated all age group to get heard immunity.
China	Han et al. [68]	A data-driven mechanistic model with five compartments.	Seventeen age group are considered to explore the time varying vaccination effect.	A time varying vaccination program for the different age groups is the most effectively way for reducing deaths and infections. Early phase of high vaccination capacity is the key to achieve great advances of policies arrangements.	To minimize the number of deaths and ICU admissions, over 65 years older people and near of them should be vaccinated before moving to other groups.
UK	Moore et al. [69]	A modified SEIR-type model with force of infection determines by age dependent social contact matrices.	New secondary infections increase due to the first infections within a household. Secondary household contacts to be quarantined and subsequently performance no additional role.	Vaccine is most effective for elderly and vulnerable population which reduce number of deaths and healthcare demands.	To reduce death and health care demand elderly people must be vaccinated.
UK	Moore et al. [70]	Age-structured mathematical model	Incorporated two-dose vaccination and non-pharmaceutical interventions to explore the different scenarios.	vaccination alone is not sufficient to contain the outbreak of COVID-19. In the absence of non-pharmaceutical intervention, the vaccine will prevent 85% infections of the population.	Combine vaccination and non-pharmaceutical interventions is essential to eliminate COVID-19 outbreak in the UK.

**Table 3:** Review of key literature for other infectious diseases modelling.

Author(s)	Research Aims	Methodology	Significant Findings
Kanyiri et al. [71]	Mathematical modelling of the transmission dynamics of influenza.	Dynamical systems, analysis of stability of stationary points, sensitivity analysis.	A mathematical model incorporating the aspect of drug resistance is formulated. The qualitative analysis of the model is given in terms of the control reproduction number, $R_{c}$. Numerical simulations reveal that despite reducing the reproduction number below unity, influenza can still persist in the population. Hence, it is essential, in addition to vaccination, to apply other strategies to curb the spread of influenza.
Wu et al. [72]	Modelling of univariate and multivariate time series data.	Transformer-based machine learning.	The authors developed a novel method which uses transformer-based machine learning models to forecast time series data. This approach works by leveraging self-attention mechanisms to learn complex patterns and dynamics from time series data. Their framework can be applied to both univariate and multivariate time series data. The authors used influenza-like illness (ILI) forecasting as a case study and showed that their transformer-based model can accurately forecast ILI prevalence using a variety of features.
Lewnard et al. [73]	Assessment of the effectiveness of interventions used in the Ebola outbreak and how these interventions may be used individually or in combination to avert future Ebola Virus Disease (EVD) outbreaks.	Building of a transmission model for the Ebola outbreak fitted to Ebola cases and deaths in Montserrado, Liberia. The model was used to assess the intervention measures such as expanding EVD treatment centres, allocation of PPE and case ascertainment numbers. September 23, 2014 was used as the base for all behaviour and contact patterns. The primary outcome measure was the expected number of cases averted by December 15, 2014.	The authors estimated that the reproductive number for EVD in Montserrado was 2.49. The allocation of 4800 additional beds at EVD treatment centres and increasing case ascertainment numbers 5-fold can avert 77,312 cases by December 15, 2014.
Kucharski et al. [74]	To understand the transmission dynamics of Zika virus (ZIKV) using a mathematical model of vector-borne infections.	A compartmental mathematical model was used to simulate vector-borne transmission. People and mosquitoes were modelled using a susceptible-exposed-infectious-removed (SEIR) framework.	An estimation of key epidemiological parameters such as the reproduction rate. Median estimates of 2.6–4.8 reproduction rates were found. An estimated 94% of the total population of the 6 archipelagos of French Polynesia were found to be infected during the outbreak. Based on the demography of French Polynesia and the results, an implication was that an initial ZIKV infection provided protection against future infections. It would also take between 12–20 years before there was a sufficient number of susceptible individuals for ZIKV to re-emerge.
Farah et al. [75]	To develop an efficient, computationally inexpensive Bayesian dynamic model for influenza.	A statistical model that combines a Gaussian process (GP) for the output function of the simulator with a dynamic linear model (DLM) for its evolution through time was developed.	The modelling framework is found to be both flexible and tractable, resulting in efficient posterior inference for the parameters of the influenza epidemic.
Luksza and Lassig [76]	To build a model to predict the evolution of the influenza virus for vaccine selection.	Sequence data which contain HA (a particular type of protein) were used to build genealogical trees. Strain frequencies were then estimated, and mutations were mapped. Predictions were done based on the model fitted. Based on the results, a vaccine strain was selected.	Factors that determine the fitness of a strain were found. A principled method for vaccine selection was suggested.
Agusto and Khan [77]	To investigate the optimal control strategy for curtailing the spread of dengue disease in Pakistan.	Optimal control theory is used to compare the different intervention strategies, including insecticide use and vaccination.	The results show that a strong reciprocal relationship exists between the insecticide use and vaccination. The cost of insecticide increases as the use of vaccination increases. Due to the increase in cost, the use of insecticide slightly increases when vaccination decreases.
Kuddus et al. [78]	To estimate the drug-resistant tuberculosis amplification rate and intervention strategies in Bangladesh.	Optimal control strategy is used to evaluate the cost-effectiveness of varying combinations of four basic control strategies—distancing, latent case finding, case holding and active case finding.	The results reveal that a combination of one or more intervention strategies is the most cost-effective way for controlling the outbreak of drug-susceptible and multi-drug resistant tuberculosis in Bangladesh.
Rahman and Kuddus [79]	To support the National Malaria Control Program for the design and characterisation of the malaria disease in Bangladesh.	A reliable qualitative and quantitative modelling technique used to identify the most influential factors in the outbreak of malaria.	From a qualitative viewpoint, the results show that service factors, disease related factors, environmental factors, and sociological factors are significant. From the quantitative modelling approach, the results reveal that the transmission rate is the most important risk factor for the outbreak of malaria in Bangladesh.
Bhunu et al. [80]	To assess the effects of smoking on the transmission dynamics of tuberculosis.	A transmission dynamics of tuberculosis model was used, considering the fact that some people in the population are smoking in order to assess the influence of smoking on tuberculosis transmission.	The results reveal that smoking enhances tuberculosis transmission and progression from latent tuberculosis cases to active tuberculosis cases. This study also shows that the number of active tuberculosis cases increases as the number of smokers increases.

## Data Availability

Not applicable.
